# Gabapentin Decreases Narcotic Usage: Enhanced Recovery after Surgery Pathway in Free Autologous Breast Reconstruction

**DOI:** 10.1097/GOX.0000000000002350

**Published:** 2019-08-08

**Authors:** Kenneth L. Fan, Kyle Luvisa, Cara K. Black, Peter Wirth, Manas Nigam, Rachel Camden, Dong Won Lee, Joseph Myers, David H. Song

**Affiliations:** From the * Department of Plastic and Reconstructive Surgery, MedStar Georgetown University Hospital, Washington, D.C.; † Georgetown University, School of Medicine, Washington, D.C.; ‡ Department of Plastic and Reconstructive Surgery, Yonsei University College of Medicine, Seoul, Korea; § Department of Anesthesia, MedStar Georgetown University Hospital, Washington D.C.

## Abstract

The opioid crisis is public health emergency, in part due to physician prescribing practices. As a result, there is an increased interest in reducing narcotic use in the postsurgical setting.

**Methods:**

From January 1, 2018, to October 31, 2018, we employed a multidisciplinary, multimodal Enhanced Recovery After Surgery (ERAS) pathway abdominally based free tissue transfer involving the rectus. Preoperative, intraoperative, and postoperative nonnarcotic modalities were emphasized. Factors in reducing narcotic consumption, pain scores, and antiemetic use were identified.

**Results:**

Forty-two patients were included for a total of 66 free flaps, with a 98.4%(65/66) success rate. Average postoperative in-hospital milligram morphine equivalent (MME) use was 37.5, but decreased 85% from 80.9 MME per day to 12.9 MME per day during the study period. Average pain scores and antiemetic doses also decreased. Postoperative gabapentin was associated with a significant 59.8 mg decrease in postoperative MME use, 21% in self-reported pain, and a 2.5 fewer doses of antiemetics administered but increased time to ambulation by 0.89 days. Postoperative acetaminophen was associated with a significant 3.0 point decrease in self-reported pain.

**Conclusions:**

This study represents our early experience. A shift in the institutional mindset of pain control was necessary for adoption of the ERAS protocol. While the ERAS pathway functions to reduce stress and return patients to homeostasis following surgery, postoperative gabapentin resulted in the greatest reduction in postoperative opioid use, self-reported pain, and postoperative nausea vomiting compared to any other modality.

## INTRODUCTION

Enhanced recovery after surgery (ERAS) was described by Denmark surgeon, Henrik Kehlet, in 1997.^1^ ERAS protocols employ a multimodal, multidisciplinary approach to surgical patient care that aims to decrease perioperative stress, increase quality of care, and expedite recovery. Various core elements of ERAS care throughout the preadmission, preoperative, intraoperative, and postoperative process are critical to success.^2^ The ERAS pathway was initially used in the setting of colorectal surgery, but has been shown to improve outcomes in many other surgical specialties.^2–7^ In microsurgical breast reconstruction, the ERAS pathway reduces pain scores, opioid use, and length of stay, resulting in significant cost savings, without increasing complication rates compared with traditional postoperative care.^8–12^ Furthermore, ERAS strategies result in earlier mobilization, decreased nausea, and increased patient satisfaction.^13,14^

Such discussion is highly relevant in light of the opioid epidemic facing the United States.^15–17^ Spurred by the introduction of pain as a fifth vital sign and fueled by pharmaceutical companies, sales of oxycodone and methadone quadrupled between 1997 and 2002.^18^ Opioid-related deaths are at an all-time high with 115 Americans dying each day from overdoses in 2016; 40% of these deaths are the result of prescription opioids.^15^ The US Department of Health and Human Services has declared the opioid crisis a public health emergency making reevaluation of postsurgical prescribing practices critical at this juncture.^17^

Although it has been well established that the benefits of ERAS protocols for microvascular breast reconstruction are manifold,^8–12^ the contribution of each component within the ERAS pathway in decreasing narcotic usage remains to be deciphered.^10,11^ Most studies attribute the success of the protocol to the synergistic multimodal therapies bestowing homeostasis after surgical stress, without delineating the effects of each individual item.^10,11^ We present our experience in implementing our ERAS protocol in autologous breast reconstruction patients and the effect on opioid use, pain scores, and doses of antiemetic medications. Furthermore, we seek to examine the contribution of each modality within the ERAS protocol to reduce narcotic usage and postoperative nausea vomiting. Understanding the components critical in reducing postoperative opioid use will enhance the application of ERAS protocols.

## METHODS

Over the past year (January 1, 2018, to October 31, 2018), our ERAS protocol was employed through coordination with surgical teams, anesthesia teams, and nursing staff. A retrospective chart review was performed for 47 patients with the new protocol in place who underwent either unilateral, or bilateral breast reconstruction with muscle sparing transverse rectus abdominis muscle (MS-TRAM) or deep inferior epigastric artery perforator (DIEP) flaps with IRB approval (MHRI #2018–173). Patients receiving only superficial inferior epigastric artery (SIEA) and the vertical upper gracilis flaps were eliminated from analysis. All operations were conducted by the same surgeon at a single institution. Demographic information was collected, and medications they received preoperatively, intraoperatively, postoperatively, and upon discharge from the hospital.

### Perioperative Care and ERAS Protocol

**Fig. 1.**
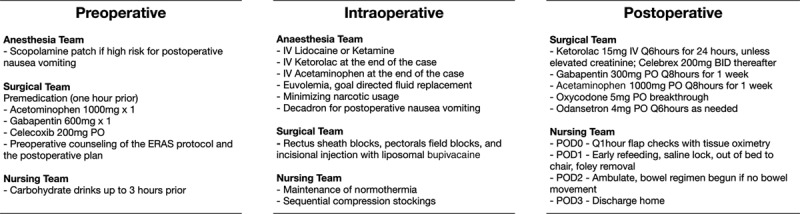
ERAS protocol employed. IV, intravenous; PO, postoperative.

The current iteration of our ERAS protocol for microvascular breast reconstruction was developed by the multidisciplinary coordination between our anesthesia, surgical, and nursing teams (Fig. 1). Preoperatively, educational materials, and counseling were provided to patients describing the ERAS protocol, flap reconstruction, and a detailed description of the postoperative plan. In the preoperative setting, patients were premedicated with gabapentin, acetaminophen, and celecoxib. Medications were not prescribed in the setting of allergies, or medical contraindications (eg, elevated creatinine or history of gastrointestinal bleed). Patients were screened for postoperative nausea and vomiting, and given preoperative scopolamine patches and intraoperative dexamethasone. Intraoperatively, anterior rectus sheath block, pectoralis field block, and incisional local anesthesia particularly at drain sites was provided using liposomal bupivacaine diluted with saline. Anesthesia teams administered intravenous lidocaine or ketamine drips and intravenous acetaminophen (1,000 mg) and ketorolac (30 mg) at the end of the case. Narcotics were used sparingly. Drain burden was minimized by using a single French drain and split at the flute in abdominal closures.^10^

Postoperatively, a standardized recovery regimen was initiated with a clear understanding of surgical milestones between the patient, nursing staff, and surgical teams. Visually highlighting these milestones in the patient rooms have been helpful in encouraging participation. Patients were given around the clock intravenous ketorolac for 24 hours (unless contraindicated) followed by per oral (PO) celecoxib, PO acetaminophen, and/or PO gabapentin. Narcotics were provided only for breakthrough pain. Doppler checks were not employed. Instead, nursing staff checked tissue oximetry (Vioptix, Newark, Calif.), and alerted surgical teams during acute drops.^19^ Patients were discharged with the same nonnarcotic analgesics as given in hospital along with 5–10 tablets of narcotic (oxycodone 5 mg) for severe pain.

### Statistical Analysis

All data were compiled into Microsoft Excel. The data were organized by patient. Opioid medications were coded separately, and subsequently converted to milligram morphine equivalents (MMEs) to standardize opiate use across patients. The total amount of MME was divided by the length of stay to give the final outcome variable of average daily MME for each patient. Average pain throughout the hospital stay was averaged by nursing records of patients’ self-reported pain—given on an increasing 10-point scale. The number of antiemetic doses (4 mg ondansetron or 12.5 mg promethazine) given during the hospital stay was summed in total.

Student’s *t*-test was used to compare differences between means. Linear regression was used to determine which ERAS medications correlated to the 4 major outcomes of interest: daily MME use, average pain, antiemetic use, and length of hospital stay. The 4 major outcomes of interest were converted into binary variables, and Fischer’s exact test was used to determine odds ratios from 2 × 2 contingency tables for the ERAS medications and all 4 of these variables. A 2-tailed value of *P* <0.05 was considered statistically significant, and 95% confidence intervals are reported. All statistical analyses were conducted using IBM SPSS Statistics for Macintosh, version 25.

## RESULTS

Forty-two patients were included in the analysis, with a total of 66 flaps (33 MS-TRAMs, 31 DIEP, 2 SIEAs). The 2 SIEA flaps included in the analysis had an MS-TRAM on one side. Five patients were eliminated from analysis due to having only SIEA flaps. Twenty-three patients had immediate reconstructions, and 19 were delayed reconstructions. All operations were conducted by the senior surgeon at a single institution. Of the operative complications(9.5%, 4/42), one patient undergoing bilateral MS-TRAM suffered a flap failure POD6 after discharge due to undiscovered hypercoagulability disorder before surgery (Factor V Leiden) for a 98.4% (65/66) flap success rate. Another unilateral DIEP was salvaged after a take back for venous insufficiency POD1 detected on tissue oximetry. One patient required intraoperative evacuation of a hematoma that developed underneath her flap POD3 while ambulating. Another patient required interventional radiology (IR) drainage of an abdominal wall seroma after drains were removed POD17. The incidence of breast delayed wound healing was 11%, abdominal delayed wound healing was 14%, seroma rate was 2%, hematoma rate was 3%, and incidence of cellulitis was 3%.

BMI (*P* = 0.029) and the number of flaps (*P* = 0.031) performed were statistically significantly correlated with abdominal wound healing complications. Smoking was significantly associated with seroma (*P* = 0.000) and infection (*P* = 0.000). There was no association between administration of ERAS medications and surgical complications (*P* > 0.05). There were 2 visits to the emergency room after discharge, one for abdominal seroma drained by interventional radiology the following day, and one for pulmonary embolism despite home prophylactic enoxaparin. There were no emergency room visits for uncontrolled pain.

### Effect of ERAS on the Study Population

**Fig. 2.**
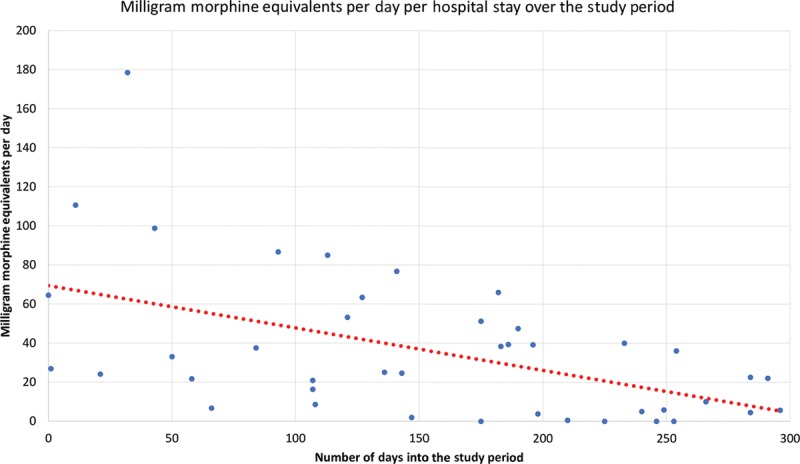
MME per day per hospital stay.

**Fig. 3.**
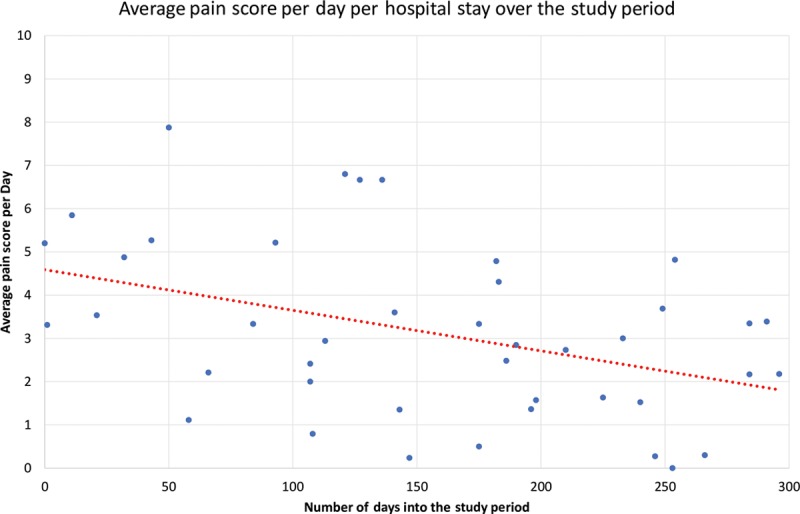
Average pain score per day per hospital stay.

**Fig. 4.**
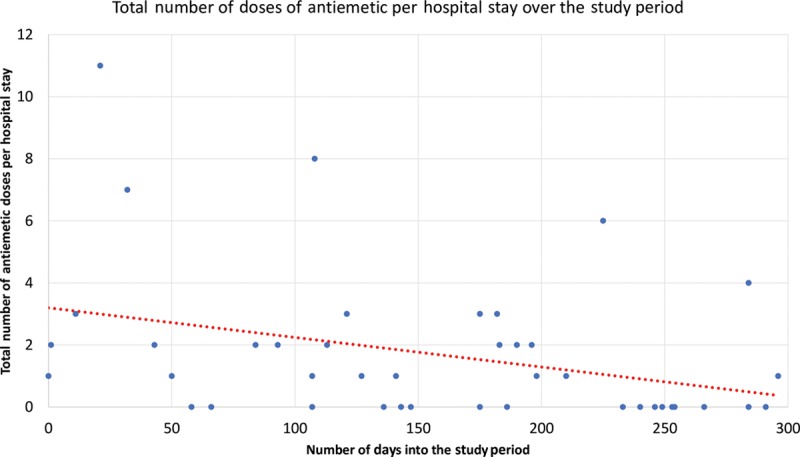
Total doses of antiemetic per hospital stay.

Forty-three percent of patients received all ERAS (gabapentin, acetaminophen, and celecoxib) medications preoperatively. Intraoperatively, liposomal bupivacaine was used in 83% of cases for a rectus sheath and incision block. Eighty-five percent of patients received either intravenous lidocaine or ketamine. Intravenous acetaminophen was administered in 95% of patients. Intravenous ketorolac was administered 21% of the time. Intraoperative narcotics were used in all cases, either fentanyl or hydromorphone. Average MME use per case was 134.8 mg. Eighty-three percent of patients received all ERAS medications postoperatively. Average MME use per day was 35.7 for this cohort, but decreased significantly throughout the study period (Fig. 2). The first 5 patients averaged 80.9 MME per day, whereas the last 5 patients averaged 12.9 MME per day. Average pain scores decreased significantly throughout the study period (Fig. 3). Total antiemetic doses on average was 1.7 per hospital stay but decreased throughout the study period (Fig. 4). The first 5 patients averaged 4.8 antiemetic doses per hospital stay, whereas the last 5 patients averaged 1 antiemetic dose per hospital stay. On average, patients’ diets were resumed, and they were out of bed to chair by POD1, and ambulating by POD2. Average hospital stay was 3.8 days in this study period. There was no difference between DIEP versus MS-TRAM flaps or immediate versus delayed reconstruction with regards to average pain scores, days to ambulation, antiemetic use, hospital stay, postoperative MME use, or complications (*P* > 0.05).

### Effect of Individual Components

Linear regression demonstrated use of all preoperative (gabapentin, acetaminophen, and celecoxib) and intraoperative (lidocaine, ketamine, and liposomal bupivacaine) ERAS medications which did not result in significant change in examined measures (Table 1). The use of all postoperative (gabapentin, acetaminophen, ketorolac ± celecoxib) ERAS medications overall decreased postoperative MME by 53.2 (*P* = 0.004; B = −53.246, −87.82; −18.68), decreased average self-reported pain by 2.47 points (*P* = 0.018; B = −2.47, −4.49; −0.44), but increased the number days to ambulation by 0.837 days (*P* = 0.014, B = 0.937, 0.20; 1.68). Linear regression was also run using all ERAS medications as independent variables to determine which medications had the greatest effect on postoperative opioid use, average self-reported pain while in the hospital, antiemetic use while in the hospital, and average hospital stay (Table 2). The use of gabapentin postoperatively was associated with a 59.8-mg decrease in average postoperative MME use per day (*P* = 0.001; B = −59.83, −93.36; −26.30); a 2.1-point decrease (on a 10-point scale) in average self-reported pain while in the hospital (*P* = 0.031; B = −2.11, −4.01; −0.21); and a 2.5 dose decrease in the number of antiemetic doses given during hospital stay (*P* = 0.045; B = −2.48, −4.88; −0.056). However, gabapentin was associated with a 0.89-day increase in days to ambulation (*P* = 0.029, B = 0.892, 0.10–1.68). Preoperative and postoperative acetaminophen use was also associated with a 2.9 (*P* = 0.045; B = −2.9, −5.703; −0.66) and 3.0 (*P* = 0.027; B = −3.03, −5.69; −0.37) point decrease, respectively, in average self-reported pain while in the hospital.

**Table 1.** The Effect of ERAS Medications on Pain, Nausea, Length of Stay, and Ambulation Variables

	Postoperative MME				Average Pain				No. Antiemetic Doses				Length of Stay				Days to Ambulation			
	*P*	B	CI		*P*	B	CI		*P*	B	CI		*P*	B	CI		*P*	B	CI	
Preoperative ERAS	0.436	−8.544	−30.5	13.45	0.655	−0.286	−1.573	1.002	0.807	−0.19	−1.755	1.375	0.544	−0.208	−0.9	0.483	0.785	−0.064	−0.539	0.41
Postoperative ERAS	0.004	−53.25	−87.8	−18.68	0.018	−2.466	−4.489	−0.442	0.075	−2.224	−4.684	0.237	0.511	−0.355	−1.442	0.731	0.014	0.937	0.199	1.676

**Table 2.** The Effect of Specific ERAS Medications on Pain, Nausea, Length of Stay, and Ambulation Variables

	Postoperative MME			Average Pain			Antiemetic Doses			Length of Stay			Days to Ambulation		
	*P*	B	CI	*P*	B	CI	*P*	B	CI	*P*	B	CI	*P*	B	CI
Preoperative gabapentin	0.464	16.683	(−29.302 to 62.669)	0.131	−1.976	(−4.579 to 0.627)	0.593	0.872	(−2.435 to 4.178)	0.947	0.05	(−1.483 to 1.583)	0.298	0.476	(−0.45 to 1.4)
Preoperative acetaminophen	0.364	−22.442	(−72.240 to 27.356)	0.045	−2.884	(−5.703 to −0.066)	0.672	0.748	(−2.833 to 0.4328)	0.994	0.006	(−1.653 to 1.666)	0.472	−0.359	(−1.37 to 0.65)
Preoperative celecoxib	0.836	4.165	(−36.763 to 45.092)	0.243	−1.35	(−3.666 to 0.966)	0.204	−1.867	(−4.810 to 1.076)	0.95	0.042	(−1.3222 to 1.406)	0.555	−0.241	(−1.07 to 0.59)
Preoperative aspirin	0.414	−14.895	(−51.726 to 21.937)	0.874	−0.163	(−2.247 to 1.922)	0.84	0.264	(−2.384 to 2.912	0.758	−0.187	(−1.414 to 1.041)	0.122	−0.572	(−1.31 to 0.16)
Intraoperative lidocaine	0.613	−6.666	(−33.368 to 20.035)	0.543	0.454	(−1.057 to 1.965)	0.434	−0.744	(−2.664 to 1.176)	0.903	−0.053	(−0.943 to 0.837)	0.652	0.118	(−0.42 to 0.65)
Intraoperative ketamine	0.688	−7.108	(−42.975 to 28.759)	0.942	−0.073	(−2.103 to 1.957)	0.713	0.468	(−2.111 to 3.047)	0.513	0.387	(−0.808 to 1.582)	0.799	0.094	(−0.66 to 0.85)
Intraoperative ketorolac	0.134	−24.832	(−57.822 to 8.159)	0.638	0.433	(−1.434 to 2.3)	0.711	−0.434	(−2.806 to 1.938)	0.367	0.492	(−0.607-1.592)	0.045	0.68	(0.02 to 1.34)
Intraoperative acetaminophen	0.277	−29.218	(−83.170 to 24.734)	0.347	−1.426	(−4.479 to 1.628)	0.7	0.737	(−3.143 to 4.616)	0.255	1.019	(−0.779 to 2.817)	0.243	0.626	(−0.45 to 1.71)
Intraoperative liposomal bupivacaine	0.77	−5.154	(−40.951 to 30.643)	0.46	0.74	(−1.286 to 2.766)	0.717	−0.461	(−3.034 to 2.113)	0.194	0.776	(−0.417 to 1.969)	0.308	0.372	(−0.36 to 1.11)
Postoperative ketorolac (IV)	0.188	22.659	(−11.734 to 57.052)	0.593	0.513	(−1.433 to 2.460)	0.511	−0.803	(−3.276 to 1.67)	0.129	−0.876	(−2.022 to 0.270)	0.115	0.547	(−0.14 to 1.24)
Postoperative gabapentin	0.001	−59.828	(−93.361 to −26.296)	0.031	−2.108	(−4.006 to −0.210)	0.045	−2.467	(−4.878 to −0.056)	0.465	−0.404	(−1.522 to 0.713)	0.029	0.892	(0.10 to 1.68)
Postoperative acetaminophen	0.17	32.359	(−14.666 to 79.384)	0.027	−3.028	(−5.689 to −0.366)	0.221	2.067	(−1.314 to 5.448)	0.84	−0.156	(−1.723 to 1.411)	0.223	−0.574	(−1.52 to 0.37)
Postoperative celecoxib	0.497	−9.166	(−36.457 to 18.126)	0.681	−0.314	(−1.858 to 1.231)	0.245	−1.137	(−3.100 to 0.825)	0.872	−0.072	(−0.982 to −0.837)	0.532	0.166	(−0.37 to 0.71)

To further analyze the effect of gabapentin, bivariate analysis was performed to calculate the odds of MME average less than 50 mg per day, less than 5 antiemetic doses, average self-reported pain less than 5, length of hospital stay ≤3 days, and ambulation by POD2 after postoperative gabapentin use were calculated (Table 3). Giving postoperative gabapentin increased the odds of a patient receiving less than 50 mg of oral morphine equivalent per day by 8.3 times (*P* = 0.0321, OR = 8.286, 1.255–54.707), and the odds of average self-reported pain below 5 by 16 times (*P* = 0.0079, 16, 2.186–117.094). The use of gabapentin in the postoperative period was not associated with the increased the odds of a patient requiring less than 5 antiemetic or length of stay (*P* > 0.05).

**Table 3.** Effect of Gabapentin on Pain, Nausea, Length of Stay, and Ambulation Variables

Gabapentin			
	Significance (*P*)	OR	CI
**Odds of <50 MME**	**0.0321**	**8.286**	**1.255–54.707**
**Odds of Pain <5**	**0.0079**	**16**	**2.186–117.094**
Odds of <5 Antiemetic doses	0.091	8.5	.926–78.0261
Length of stay ≤3 d	0.673	1.79	0.29–11.04
Ambulation later than POD2	0.86	0.81	0.08–8.42

## DISCUSSION

The ERAS protocol has been shown to be beneficial in multiple forms of breast surgery, including prosthetic- and autologous-based reconstruction.^8–12^ Key components include preoperative counseling, optimization of nutrition, standardization and pain control modalities, and early mobilization.^20–23^ Batdorf et al^10^ first described ERAS use in microvascular breast reconstruction, finding decreased length of stay (3.9 days), opioid usage (to 55.6 MME per day), and pain scores. Although our length of stay was similar (3.8 days), our narcotic usage was lower overall (35.7 MME per day), and dramatically lower in the last 5 patients because employment of our ERAS protocol (12.9 MME per day). Astanehe et al similarly found a 13 MME per day average narcotic requirement after full application of their ERAS protocol, with a 4.8 day length of stay in a cohort of 330 microvascular breast reconstructions.^3^

Because of the reduction in narcotic use, decreased antiemetic requirement, decreased length of stay, and cost savings, several authors have called for ERAS to be the standard of postoperative care in microsurgical breast reconstruction.^8,9^ However, the main narcotic reducing components of the pathway remain to be understood.^10,11^ Some applications are prohibitive due to clinical restrictions; for example, not all institutions have liposomal bupivacaine available.^11^ Therefore, distilling the key elements of the ERAS pathway is critical to understanding its significance. This is the first study to identify postoperative gabapentin as critical in significantly reducing average postoperative MME use per day by 59.8 mg, a 21% in pain scores, and a reduction of 2.5 doses of antiemetics during the hospital stay after microvascular breast reconstruction. Gabapentin significantly increased odds by 8.3 times that patients would receive less 50 mg MME per day, and 16 times increased odds of average pain scores below 5. Acetaminophen significantly reduced self-reported pain scores by an average of 3.0 points. These findings were similar to that of Barker et al,^24^ who demonstrated significantly decreased postanesthesia narcotic use with multiple preoperative ERAS medications (gabapentin, acetaminophen, and celecoxib) in outpatient breast surgery.

Gabapentin works through mitigating neuropeptide release by binding voltage-sensitive calcium channels within cortical and dorsal horn neurons.^25^ In large meta-analyses, gabapentin has been associated with significantly decreased narcotic usage and pain scores.^26,27^ At 24 hours, Mishriky et al^26^ found an 8.28 MME decrease, but an increase in sedation, dizziness, and visual disturbance at 24 hours. In a double randomized controlled trial, a single dose of 600 mg gabapentin administered before mastectomy was associated with significantly decreased pain scores and morphine consumption postoperatively, without significant differences in dizziness, nausea/vomiting, or blurred vision.^28–30^ Gabapentin was associated with an increase in days until ambulation on linear regression for our cohort. Studies have generally found postoperative gabapentin more efficacious than a single preoperative loading dose, as our results confirm.^31,32^

Because ERAS protocol implementation, we have observed a dramatic and sustained decrease in narcotic usage, pain scores, and antiemetic use. Detailed preoperative and postoperative counseling of the postoperative recovery course was critical in shifting the culture of pain control and decreasing narcotic usage.^13^ Buy-in from nursing staff, anesthesia providers, residents, and other advanced practitioners were necessary in effecting these changes.^10^ Although the surgically directed components of our ERAS protocol were consistent and showed significant positive effect, future collaborative efforts need to be directed at reduction in intraoperative MME. Prior, liberal use of narcotic analgesia was fueled by emphasis of pain as a fifth vital sign, and concerns over Press–Ganey scores.^18^ However, when explained, patients anticipate nonnarcotic pain control and make mental preparations for surgical milestones, such as out of bed to chair, ambulating, and going home. With education by the surgical team, perioperative narcotic usage decreased over time. Upon discharge, we observed no return to the emergency room for additional pain medication. Anecdotally, patients did not request them in office either.

Although microvascular breast reconstruction has been historically viewed as a major operation at our center, enacting this protocol has shifted the institutional mindset, similar to that described by Bonde et al.^33^ In their examination of ERAS protocol in microvascular breast reconstruction, a plan involving nursing staff was critical in reducing overall pain scores, and length of stay from 6.2 days to 72 hours. Additionally, eliminating disruptive Q1 hour doppler checks and relying on tissue oximetry has been instrumental in helping patients get rest. Placing patients on a surgical floor, instead of a loud intensive care unit has also helped in that regard. Although the ERAS protocol did not significantly change length of stay in our cohort, our length of stay was low at 3.8 days.^9^

In this cohort, transversus abdominis plane (TAP) blocks were not performed. Instead, we perform rectus sheath blocks with liposomal bupivacaine, which on regression was found not to be significant in reducing MME usage, pain scores, number of doses of antiemetics, time to ambulation, or length of stay. Afonso et al^9^ evolved their TAP block technique from open exposure of the plane, injection by ultrasound guidance, to now with injection based on tactile feedback and a blunted needle. In that study, liposomal bupivacaine, postoperative ketorolac, shorter length of surgery, and goal-directed fluid management resulted in decrease in opioid use and length of stay. Gabapentin was not used in this cohort.^9^ It is possible a properly performed TAP block with the addition of gabapentin have synergistic effects in reducing narcotic consumption. Similarly, paravertebral blocks have been demonstrated to be effective in breast reconstruction in reducing postoperative pain and length of stay, without comprising blood pressure or intraoperative fluid requirements.^34–36^ Dexamethasone was used for postoperative nausea and vomiting prophylaxis in this study.^37^ However, dexamethasone can improve pain control as well, and wider use may be warranted perioperatively in nondiabetic patients.^25,37^

Although we employ an nil per Os (NPO) period until early POD1, Astanehe et al^11^ has had success giving clear fluids immediately after surgery. In the absence of surgical complications, Batdorf et al^10^ provides patients with a solid diet on POD 0. Although intravenous lidocaine was used intraoperatively in the majority of our patients, no direct benefit was observed on regression. The majority of trials and meta-analysis demonstrating reduced pain with intraoperative intravenous lidocaine were performed in open or laparoscopic abdominal procedures.^38^ In double blind trials in mastectomy patients, intravenous lidocaine did not affect opioid consumption, postoperative nausea vomiting, or pain scores,^39^ although there is some evidence it is protective against chronic postsurgical pain.^40^

Ultimately, the retrospective, single institution nature of this examination limits the applicability of its conclusions. The application of the ERAS protocol was heterogenous in nature due to the initial learning curve and patient contraindications. Furthermore, we did not critically examine prescribing practices on follow-up. Although cost analysis was not performed, several studies have highlighted direct cost savings of ERAS protocol and liposomal bupivacaine by reducing hospital stay, cost of drugs, and unscheduled outpatient visits.^32,41–44^

## CONCLUSIONS

Full implementation of our ERAS protocol required a cultural shift in the way providers, staff, and patients viewed pain control. When applied, the ERAS protocol dramatically reduced postoperative narcotic consumption, pain scores, and antiemetic usage in our microvascular breast reconstruction patients. Although all components of the ERAS pathway function to reduce stress after surgery and achieve homeostasis, postoperative gabapentin use in our series resulted in the greatest reduction in postoperative opioid use, self-reported pain, and postoperative nausea vomiting than any other preoperative, intraoperative, or postoperative modality.
